# Understanding community antibiotic use and antimicrobial resistance in Sub-Saharan Africa: A grassroots perspective from Enugu, Nigeria

**DOI:** 10.1371/journal.pone.0353762

**Published:** 2026-07-23

**Authors:** Winifred Maduko, Emmanuel Olamijuwon, Mike Kesby, Jo Mhairi Hale

**Affiliations:** 1 School of Geography and Sustainable Development, University of St Andrews, Scotland, United Kingdom; 2 Heroes of Our Time Africa Foundation, Enugu, Nigeria; Ekiti State University College of Medicine, NIGERIA

## Abstract

Antimicrobial resistance (AMR) is a growing global health crisis, especially in low-and middle-income countries where antibiotic access is insufficiently regulated and health systems are under-resourced. In Sub-Saharan Africa, the community is often the primary site of antibiotics decision-making, shaped by informal markets, structural barriers, and cultural norms. This study investigates antimicrobial use behaviours, perceptions of AMR, and motivations for inappropriate antibiotics use and what might motivate future change, among diverse communities in Nigeria. Using a mixed-methods design, data were collected from a baseline survey (n = 1,281), 20 interviews, and 4 focus group discussions (n = 24). Antibiotic use was widespread, with 57.7% of respondents reporting use within six months, often without prescriptions and for conditions like malaria (32.3%), cough (7.6%), and fever (4.5%), many of which are non-bacterial in origin. Of those who reported antibiotic use, 48% self-medicated, sourcing antibiotics primarily from chemist shops. Misconceptions were common; only 15.7% had heard of AMR, and many confuse it with fake drugs or allergic reactions. Novel trends such as the use of antibiotics for pregnancy termination and preventive “cleansing” emerged. Structural challenges such as poverty, poor healthcare access, and lack of regulation reinforced these behaviours. Qualitative findings highlighted a deep trust in antibiotics as a “cure-all”, low health literacy, and the central role of informal drug vendors in care delivery. Participants also offered culturally embedded recommendations, including using radio jingles, community education through religious leaders, and stronger enforcement of drug regulation. This study argues that antibiotic misuse in Nigerian communities is not solely a function of ignorance but a rational response to systemic healthcare limitations, cultural logics, and informal care networks. Tackling AMR in this context requires inclusive, community-informed interventions that address both behavioural and structural determinants of misuse. The study offers actionable insights for AMR policy, communication strategies, and community engagement in Sub-Saharan Africa.

## Introduction

Antimicrobial resistance (AMR) is one of the most pressing global public health threats of our time. The World Health Organization warns that without urgent and coordinated action; we may be entering a ‘post-antibiotic era’ in which common infections and minor injuries may become difficult to treat or even fatal [1]. While AMR is a global concern, its implications are particularly serious in Low-and Middle-Income countries (LMICs), especially in Sub-Saharan Africa, where health systems are often under-resourced, health and diagnostic infrastructures are weak, and antibiotics are frequently (mis)used [2–5]. In many African settings, communities often become a primary site for decisions about accessing and using antibiotics, largely because informal markets, cultural beliefs, economic pressures, and mistrust in healthcare providers limit reliance on formal health facilities [6]. Antibiotics are frequently purchased from chemist shops or street vendors, often without prescriptions, sometimes in therapeutically inappropriate ways, and/or used for viral and parasitic infections for which they are ineffective [7–10]. Such practices, coupled with the largely unregulated availability of antibiotics, create fertile ground for the emergence and spread of resistant pathogens, leading to adverse drug reactions, economic losses, and prolonged illness.

Although the burden of AMR is rising across Sub-Saharan Africa [11,12], its dynamics in the region are shaped by social, cultural, and structural factors. Studies highlight how weak regulatory frameworks and unregulated antibiotic availability contribute to widespread misuses across the region [5,12]. Hospital and patient-based studies in Ghana and Nigeria show high antibiotic consumption but emphasize stewardship largely within these hospital/formal facilities [13–15], leaving accounts of community-level practices underexplored. These studies provide important insights into prescribing practices and facility-based stewardship, however, because much antibiotics use exists beyond those spaces, continuous research on antibiotic use in everyday community contexts is necessary. Meanwhile, prescription audits in outpatients and retail pharmacy-based surveys across countries like Kenya, Uganda, and Tanzania show how antibiotics are routinely dispensed without prescription [7–11,16], highlighting concerns that antibiotics use practices are far beyond the regulations. In Nigeria specifically, existing studies have looked at perceptions of AMR and antibiotics, misconceptions and self-medication among students and health trainees [17–19], but these groups are typically younger and or more educated, which could limit the generalizability of findings to wider populations and not fully representative of wider community dynamics. Therefore, more attention needs to be paid to the dynamics of antibiotics use at the grassroots: e.g., how ordinary people currently understand illness, why they choose certain drugs, and what current social, cultural, and structural factors shape their decisions. Additionally, community level studies in other African settings indicate that antibiotic use decisions are often embedded in economic pressures, informal markets, and other factors [6,8,9]. While patterns of antibiotic use and awareness have been described in select populations, there is need for more studies that examine individuals’ decision making at the community-level on antibiotics use and the factors that shape their behaviours.

This study attempts to address these needs by examining antimicrobial use behaviours, knowledge of AMR, and antibiotic stewardship practices among diverse communities in Enugu State, Nigeria. It is informed by Andersen’s Behavioural Model of Health Services Use [20,21], which suggests that the utilization of healthcare is shaped by the interaction of ‘predisposing’ (e.g., cultural beliefs, demographic characteristics), ‘enabling’ (e.g., healthcare access, cost, informal networks), and ‘need factors’ (e.g., perceived illness severity, health system (mis)trust). Applying this theoretical lens helps to explain how antibiotic (mis)use within this study setting emerges not only from knowledge gaps but also from structural barriers, socio-cultural norms, individual health needs, economic realities and their intersections.

While prior studies have identified high rates of self-medication and misconceptions about antibiotics in settings such as Eritrea, Ethiopia, and East Africa [22–24], there remains limited evidence on the broader range of community-level antibiotic use practices, particularly those that may be informal or socially embedded. Through a large-scale baseline survey complemented by in-depth interviews and focus group discussions (FGDs), this study seeks to provide community-level insights into antibiotic use in South-Eastern Nigeria.

In distinction from previous studies, this study centres the voices of community members, highlighting their lived experiences and perspectives on AMR and antibiotic use. Furthermore, it explicitly includes persons with disabilities (PWDs), a group often marginalised in AMR research and intervention efforts, despite their heightened vulnerability to infection, increased antibiotic use, and frequent exclusion from mainstream health education initiatives. While their experiences are not extensively highlighted here, their inclusion in this study nonetheless marks an important first step, which is to include (and not exclude) them from consideration as an important social minority in AMR research, aligning with global calls for disability-inclusive health systems and equity in public health interventions [25,26,].

Through a mixed-methods design, this study seeks to answer the following questions: (1) What current antimicrobial use behaviors do community members self-report; (2) What are the prevalent patterns of misuse and for which ailments/conditions are people most likely to use antibiotics. (3) What motivates people’s current behaviours and what might motivate future changes, and their perceptions of responsibility for antimicrobial stewardship. The significance of this study lies not only in its empirical contribution but in its implications for future public health intervention design. Simply disseminating biomedical information is insufficient; effective AMR campaigns must engage with local explanatory patterns of illness, address structural access barriers, and leverage trusted community figures.

## Methods

### Ethics statement

Ethical approval for the study was obtained from the Ministry of Health Research Ethics Committee in Enugu, Nigeria (MH/MSD/REC21/617), and the School of Geography & Sustainable Development Ethics Committee at the University of St Andrews. Participants in the study provided written informed consent and received an information sheet detailing the study objectives and their participation. After going through the participant information sheet in local language, written informed consent was taken from participants by the study team.

### Study design

This study employed a mixed-methods design [27], that integrated both quantitative and qualitative approaches concurrently to enable a comprehensive understanding of antimicrobial use behaviours within communities in Enugu State, Nigeria. The justification for using this approach is the need to complement the breadth of quantitative data with the depth of qualitative insights, allowing for a more nuanced exploration of the complex drivers of antibiotic use and AMR. The quantitative component consisted of a basic descriptive baseline survey conducted across all 17 Local Government Areas (LGAs) of Enugu State, encompassing both rural and urban communities. A total of 1,281 residents participated in the baseline survey. The survey questionnaires were used to assess participants’ knowledge, attitudes, practices, and awareness related to antibiotics and AMR. The qualitative component included 20 in-depth interviews and 4 focus group discussions (FGDs) comprising 6 participants each (total n = 24), ensuring diversity in gender and age representation. The qualitative component sample size adequacy was assessed in terms of ‘information power’ [28] ensuring that the sample has enough relevant, rich and diverse information sufficient to answer the research questions. The qualitative component was designed to explore participants lived experiences, beliefs, and perceptions regarding antibiotic use, as well as motivations for current behaviours and possibilities for future behavioural change. This study was conducted between 18th July and 4th October 2024. All participants were provided with an information sheet detailing the study objectives and their role in the research. Written informed consent was obtained prior to participation in any aspect of the study.

The methods used within this study served complementary purposes: the baseline survey quantified patterns of antibiotic use, awareness, and attitudes across the state; in-depth interviews provided rich, individual-level accounts of lived experiences and decision-making processes; and FGDs highlighted collective norms, shared cultural beliefs, and group interactions that could shape antibiotic use in community settings. While some overlap is expected (e.g., certain beliefs expressed in interviews also emerging in FGDs), the group setting often generated different views, or even disagreement, and community-driven solutions were less evident in one-on-one conversations.

### Recruitment of study participants

Participant recruitment for the baseline survey was conducted in collaboration with the Enugu State Bureau of Statistics and the National Population Commission of Nigeria. As part of standard protocol for state-sanctioned surveys, the National Population Commission organizes the country into administrative units: Federal, State, Local Government Area (LGA), and Enumeration Areas (EAs). Using a systematic random sampling technique, 5 EAs were randomly selected per LGA, resulting in coverage across all 17 LGAs of Enugu State. Each Enumeration Area comprises a defined number of households. With technical support from the Enugu State Bureau of Statistics, about 76 households within the selected EAs in each LGAs were identified and approached for participation. While 5 EAs were selected per LGA, the total number of households varied between LGAs due to differences in EA size and population distribution. This multi-stage approach ensured geographic and demographic representation across both urban and rural populations in the state. A total of 1,281 participants were included in the baseline survey. The sample size was not determined through a formal power calculation because the study was primarily qualitative and not designed for inferential statistical testing. The quantitative data are presented to indicate the relative prevalence of views and patterns within the sample rather than to support inferential claims or generalisation to a wider population.

Participants for the interviews and FGDs were recruited with the support of the South-Saharan Social Development Organisation (SSDO), a non-profit organization in Nigeria with longstanding experience in community engagement and outreach within Enugu, Nigeria. In addition, recruitment was facilitated through existing networks and relationships maintained by the researchers in the area. An initial pool of participants was identified through purposive outreach, and subsequent participants were enrolled using a snowball sampling technique, wherein initial participants referred others within their social networks. The different FGDs were held in a central and accessible location in Enugu to enable participation from individuals across the different Local Government Areas in Enugu State. Overall, the study aimed to ensure diverse representation across communities in Enugu, including urban and rural residents, students, community leaders, and members of organized groups. Notably, the study intentionally included persons with disabilities (PWDs) to broaden inclusion and capture a wider diversity of voices and experiences, a critical gap in AMR research within Sub-Saharan Africa. Individuals with disabilities may face heightened vulnerability to infectious diseases and AMR due to factors such as limited access to healthcare, reduced health literacy, and higher prevalence of comorbidities [25]. Their inclusion was essential to ensure that diverse experiences, especially those often marginalized in health research, were adequately captured. All participants involved in focus group discussions were informed of the importance of confidentiality and were asked to respect the privacy of views expressed during the sessions by not disclosing or sharing information beyond the group context.

### Data collection and management

For the baseline survey, a semi-structured questionnaire was developed following a comprehensive review of relevant literature on antibiotic use, self-medication practices, and antimicrobial resistance in Sub-Saharan Africa [7,8,22,23]. The instrument underwent an assessment for content validity, structural clarity, and overall comprehensibility by the research team. A pilot test was conducted with research enumerators who later supported data collection. This pilot was integrated into a training workshop held in Enugu to ensure quality and consistency in administering the tool across diverse communities. The final version of the questionnaire was digitized using an open-access data collection and management platform. It covered key themes related to antimicrobial drug use, awareness, and behaviours (**see** **S1 Text** for the final questionnaire). All responses were anonymised, and informed consent was obtained from participants before data collection.

For the interviews and FGDs, data were collected using a structured guide developed through extensive literature review and informed by insights from similar studies [24]. The guide covered broad thematic areas, including experiences of illness, pathways to care, antibiotic access and use, healthcare experiences, perceptions of AMR, awareness and misconceptions, stewardship practices, and public health communication. Data were collected through audio recordings and fieldnotes, and subsequently transcribed verbatim into Microsoft Word. All interview and FGD data were anonymised. Participants received information sheets and provided written informed consent prior to participation.

### Data analysis

Quantitative data from the completed questionnaires were extracted into Microsoft Excel and analysed using descriptive statistics. Frequencies and percentages were used to summarise responses to closed-ended questions, with selected data visually presented in tables to aid interpretation. Open-ended responses were qualitatively reviewed to identify recurring ideas and perspectives. These qualitative insights were thematically grouped and integrated into the results to enrich the interpretation of survey findings.

Qualitative data from interviews and FGDs were analysed using Braun and Clarke’s six-step thematic analysis framework [29,30]. This involved familiarisation with the data, generating initial codes, searching for themes, reviewing themes, defining and naming themes, and producing the report. Transcripts were read line-by-line, and segments of text were coded inductively using NVivo (version 10). Recurrent keywords, sentence patterns, and meaningful fragments were grouped into preliminary codes, which were then refined through a process of merging, collapsing, or renaming to remove redundancy and improve coherence. Final themes reflected participants’ perceptions, lived experiences, and contextual understanding of antibiotic use, misuse, and AMR. These are presented in the results section with illustrative quotes and insights. (**See** **S1 Appendix** for the final Nvivo codebook).

### Definition of key variables

Antibiotic misuse in this study refers to the use of antibiotics in ways that deviate from biomedical recommendations. This includes (i) use without prescription, (ii) use for non-bacterial conditions (e.g., malaria, viral infections), (iii) incomplete dosage or premature discontinuation, and (iv) use for non-medical purposes. These were explored qualitatively through participants’ narratives of use practices and measured quantitatively through survey indicators (e.g., self-medication rates, conditions treated, treatment completion). AMR awareness refers to participants’ recognition and understanding of antimicrobial resistance as a concept. Qualitatively, awareness was explored through participants’ descriptions of drug effectiveness, resistance, and related misconceptions. Quantitatively, this was assessed through survey questions on whether participants had heard of AMR and their ability to correctly identify its meaning.

### Inclusivity in global research

Additional information regarding the ethical, cultural, and scientific considerations specific to inclusivity in global research is included in the Supporting Information (**S1 Checklist**).

## Results

In this mixed methods study, we take an integrated approach, displaying the quantitative and qualitative findings jointly, including figures, tables, and illustrative quotes from interviews and FGDs. We begin by describing the study sample, then present the findings thematically under the headings derived from the qualitative analysis.

### Descriptive profile of study participants

The baseline survey included 1,281 respondents from all 17 local government areas of Enugu State, Nigeria, capturing both urban and rural settings. **Table 1** below presents the demographic characteristics of the survey participants. Full detailed survey data tables are provided in **S1 Table**. The majority of respondents were aged 46 years and above (60%). While this shows a bias towards older age groups in our sample, in many Nigerian households, older adults often hold decision-making roles in health matters, which may have implications for antibiotic use behaviours. Gender distribution was relatively balanced, with males 663 (51%) slightly outnumbering females – 618 (48.2%). Among the recruited cohort, educational attainment was relatively low, with only 16% having tertiary education (including both undergraduate and postgraduate levels), and 19% reporting no formal education. Most respondents were self-employed (59%), reflecting a largely informal economy, while 22% were unemployed. Meanwhile, the qualitative component comprised 20 in-depth interviews and 4 FGDs. The interview participants included 9 males and 11 females, while the FGDs involved 24 participants in total, with 13 males and 11 females, ensuring a gender-balanced representation across both methods.

**Table 1:** Showing demographic characteristics of survey participants.

Response	Count	Percentage (%)
**Age Group**		
18-25	54	4.2
26-35	127	9.9
36-45	327	25.5
46-55	353	27.6
56 and above	420	32.8
**Persons with Disability**		
Yes	128	10
No	1153	90
**Education Level**		
Postgraduate education	20	1.6
Undergraduate education	184	14.4
No formal education	253	19.8
Primary school	356	27.8
Secondary school	468	36.5
**Employment Status**		
Student	12	0.9
Employed (part-time)	48	3.8
Employed (full-time)	78	6.1
Retired	86	6.7
Unemployed	293	22.9
Self-employed	764	59.6

### Perceptions and Experiences of Illness

When asked about their experiences of illness and the symptoms familiar in their community, participants explained illnesses using local language and cultural logics. Participants described symptoms as “normal” or attributed them to environmental conditions like dust or unwashed vegetables. In a few cases, healing was attributed to faith, that is faith-based interpretation for sickness. With one participant noting, *“Sometimes we just pray, and the sickness goes away.”* Some participants acknowledged prayer and divine healing as part of the healing process. One FGD participant (Participant, FGD4) stated, *“…when [they are] not responding to drugs, some people just pray for healing.”* These insights highlight that their health-seeking is not purely biomedical but mediated by culture and context. Antibiotics are part of a broader landscape of health behaviour and practice – one in which understanding, behaviour and practice are not necessarily based on scientific/medical understanding, but on habit and on ‘faith’ in cures that are believed to work.

In the qualitative findings, when asked about their experiences of illness and the symptoms familiar in their community, participants often described common symptoms (for which antibiotics were commonly used) such as persistent cough, sore throat, shortness of breath, and fever, with many attributing them to malaria, catarrh, or spiritual causes rather than bacterial infections. While these symptoms can occur with illnesses like malaria or catarrh (as identified by participants), they also overlap with bacterial respiratory infections. The confusion is not in recognising the symptoms, but in linking them to the right course of treatment. In many cases, participants used antibiotics even when their own attribution of the illness (e.g., malaria) would not warrant antibiotic use. The overlap of symptoms created diagnostic uncertainty. When people experienced throat pain or fever, they often labelled the illness simply as “sore throat” for example, a participant said: *“We just know it [this kind of sickness] as sore throat. Everyone just says, ‘I’m having sore throat.”* (Interview Participant-3, M).

### Awareness and Understanding of Antibiotics and AMR/ Knowledge and Misconceptions

In the survey, a total of 24% of participants responded ‘No’ when asked whether they know what antibiotics are. Despite the other 75% of respondents reporting awareness of antibiotics, misconceptions were widespread. While 47% of participants correctly identified antibiotics from a list of choices, as drugs that kill or inhibit bacteria, 34% believed they treat viral infections, and 15% participants believed they can treat any sickness, including “stubborn ones.” This pattern is reflected in the qualitative findings, where participants described antibiotics as ‘strong medicine’ that treats all ‘germs’, without distinguishing between bacterial and viral infections. In the qualitative findings participants in the community when talking about illness and medicine, they do not make a clear distinction between viruses and bacteria; both are conceptualized as germs or infections. Participants described antibiotics using broad terms like ‘strong medicine’ or medicine that kills germs without recognising that antibiotics specifically target bacteria and not viruses. A participant indicated that when they have flu (a viral infection) they still believe antibiotics will help, because to them it is all just ‘germs’ (Interview Participant-2, M). The categories virus and bacteria are not seen as separate. These misunderstandings reflect low antibiotic literacy.

The qualitative findings reinforce these survey patterns. Despite their confidence in antibiotic use, many participants lacked accurate understanding. Some believed antibiotics could treat viral infections or confused them with general supplements:

*“Antibiotics help build you... antibodies that fight disease.” (Interview Participant-3, M).*

Limited understanding of antibiotics was widespread. Some respondents mistook multivitamins or painkillers for antibiotics. There was also significant confusion about dosage and treatment duration:

*“People believe once the headache [symptom] stops, there is no need to take the drug again.” (Interview Participant-2, M)*. Here, the participant was describing a general practice that also applied to antibiotics use.

One participant recounted an experience where a chemist dispensed cod liver oil as an antibiotics to her, leading to complications: “…he went ahead to say that cod liver oil is antibiotics for the eye that I am not supposed to take it with the antibiotic too as it's a complication.” (Participant, FGD1). As this is a self-reported account, we cannot verify the seller’s intent or clinical reasoning; the example nevertheless shows that interactions between sellers and clients do not necessarily improve clients’ understanding of antibiotics and their intended use.

Misconceptions also included incorrect drug identification (e.g., calling cod liver oil an antibiotic, as seen above) and confusion over dosage and indications. While a few participants correctly identified antibiotics from a multiple-choice list as targeting bacteria and emphasized full-course adherence, this was the exception: *“People underuse more than misuse. They stop taking antibiotics once they feel better.” (Interview* Participant-1,M,PWD*).*

Survey data showed a similar pattern regarding understanding of AMR: 84% of participants had not heard about AMR or drug resistance, while 15% had heard of AMR, and among these, just 20% were able to select scientifically accurate definitions. The majority conflated AMR with fake drugs, allergic reactions, or spiritual factors. The qualitative findings also further reflected low awareness of antimicrobial resistance which is similar to the quantitative findings. When mentioned, it was understood simplistically as drugs “not working like before,” often blamed on fake medicine or underuse rather than overuse. It is, therefore, not surprising that participants acknowledge this gap in understanding and repeatedly identified the need for more education, both formal and informal, as central to correcting these misconceptions:

*“Education is vital. Without it, people will just go and say, ‘cut two capsules’ at the chemist.” (Interview Participant-2,M).*

### Patterns of use and source of antibiotics

The survey findings showed that antibiotic use was high, 57% of participants reported using antibiotics in the past six months. Purpose for use was driven largely by illness (75%), and preventive purposes (10%). A small fraction (2.8%) could not be clearly categorized and was grouped under “Other.” Importantly, nearly half of respondents (48%) admitted to using antibiotics without consulting a healthcare professional, a reflection of both accessibility issues and cultural normalization of self-treatment. 62% of participants primarily obtained antibiotics from chemist shops (informal drug sellers or patent medicine vendors), and a further 16% from pharmacy shops without prescriptions, while only 15% received them through prescriptions. Thus, nearly 80% of antibiotic access occurred outside formal regulation, highlighting serious gaps in antimicrobial stewardship enforcement. Qualitative accounts provide insight into these patterns of use identified in the survey, particularly the high reliance on informal sources and unregulated access. Participants relayed that access to antibiotics was mostly unregulated. Chemists frequently dispensed antibiotics without prescription, often mixing them with other drugs, a participant said: *“…I have been around chemists before. There are some chemist people that do herbal and antibiotics mixing. That is why they mix herbal with regular drugs sometimes…” (Participant, FGD2).*

The survey findings on widespread access through chemists and pharmacies without prescription are further explained by cost-related constraints highlighted in the qualitative data. Cost was also a driver of this practice: *“You know these big drugs are more expensive. The money that you have… you go down to that mixing [which is more affordable].” (Participant, FGD1).*

Another participant said*: “[…] the most important thing is money. when you come and explain what you're passing through to chemist, if you are with money, there are some drugs the person will give to you based on maybe selected by cash [the money you have]. The reason of this mixing is just because of low cash [...] chemists will cut four and another four then mix two different doses for the person because the person didn't have money to buy the complete card. By His Grace, when the person takes it first time, second time you believe. So, the most important thing is faith and try to go at the appropriate time[…]” (Participant, FGD 1).*

These qualitative insights also help to contextualize the high levels of self-medication reported in the survey. Nevertheless, participants demonstrated high confidence in antibiotics, often referring to them as “strong medicine” and using them pre-emptively or inappropriately. As one participant explained: *“…I just go and tell them to give me Azithromycin. They don’t usually ask questions... If I have sore throat or cold that lingers, I just buy azithromycin and take it.” (Interview Participant-3, M).*

### Diseases most associated with antibiotic use

When asked what illnesses they used antibiotics for, interview and FGD participants and survey respondents included malaria, typhoid, respiratory tract symptoms, and general fever as commonly treated by antibiotics. Survey participants most frequently mentioned: Malaria (239 mentions), Typhoid (128), Infection (89), Cough (56) and Fever (33). These results show that antibiotics are widely used not only for bacterial infections like typhoid but also for conditions such as malaria and cough, often viral or parasitic in origin and inappropriate for antibiotic treatment. Qualitative findings also clarified that antibiotics are considered essential for recovery regardless of illness origin. Antibiotics were frequently used for viral infections, malaria, and even non-medical purposes, such as pregnancy termination. A disturbing new finding involved the self-medicated use of antibiotics to assist with pregnancy termination. One participant noted during focus group discussion:

*“[…] as I said, antibiotics is something that can terminate something. The young girls, they believe that when they take that antibiotic and take an overdose quantity, maybe they will take three times the adult dose so that when it gets to their foetus it can flush it out. That's the belief [...] they are not using it to prevent. So, they use to terminate. So, it's not for prevention…So for some people, it works for them. For some other people, once they do tedious work consistently, they may or may not lose the baby […] yes, it will [the antibiotics] flush out the baby. So, for some people, it works for them. For some other people, once they do tedious work consistently, they may or may not lose the baby” (Participant, FGD1).*

Another added*: “I have heard that in large doses, antibiotics can flush out pregnancy.” (Interview Participant-1,M,PWD).* Respondents here describe behaviours that may have several very negative outcomes for patient health and for AMR.

### Inappropriate use and antibiotic treatment outcomes

Quantitative findings show that 49% of users stopped taking antibiotics as soon as they felt better, while only 40% completed the prescribed course. Additionally, 8% stopped once the dosage of the antibiotics they could afford finished, even if they remained unwell. Although 70% of participants felt antibiotics were effective in their recent experience, 29% reported that antibiotics had failed to cure them. However, among those who experienced failure, only 2% sought help and visited a health facility, and 1.7% turned to herbal or traditional remedies. A significant 88% gave vague or non-specific responses, suggesting uncertainty or inaction when treatment fails, with consequences that may differ depending on indication and exposure. The qualitative narratives provide context for this uncertainty, with participants recounting experiences of what they perceive were inappropriate mixtures by chemists or relying on faith when medicines seemed not to work: *“…when [they are] not responding to drugs, some people just pray for healing.”* (Participant, FGD4).

### Motivations for self-medication with antibiotics and treatment-seeking behaviours

**Fig 1:**
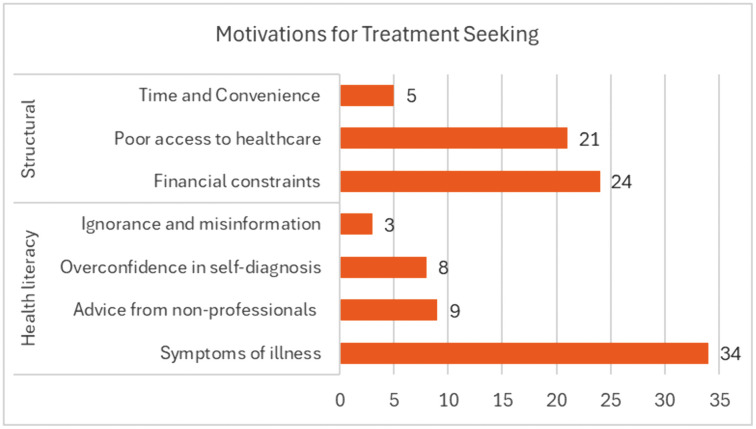
Motivation for Treatment seeking and self-medication with antibiotics among survey participants in Enugu State. (Note: Values represent the percentage (%) of respondents indicating each motivation. Percentages do not add up to 100% because participants were able to indicate more than one reason for their antibiotic use).

According to the participants responses in the survey, the motivations for their decision to take antibiotics without consulting a healthcare professional is shown in Fig 1 below.

These drivers highlight that both structural (poverty, poor access) and behavioural (social influence, misinformation) factors sustain a culture of unregulated antibiotic use. Critically, ignorance and convenience also featured, though at lower levels, pointing to gaps in health literacy and trust in the formal healthcare system. The qualitative findings further explain these survey patterns. Economic hardship, time constraints, and assumptions of self-recovery often delayed treatment-seeking as reported among participants. A common trajectory was self-medication followed by a visit to the chemist or pharmacy. Local herbal remedies (“agbo”) were widely mentioned, although trust in orthodox medicine remained strong:

“*We first take warm water and ginger to remove the mucus... but if it persists, we go for medicine.*” (Interview Participant-1, M, PWD).

Even when care was eventually sought, it followed a hierarchical process: chemist first, followed by local clinics, and hospitals only as a last resort. This reflects both economic constraints and mistrust in the health system. A participant said:

“Most people don’t go to hospital for malaria, typhoid… health centers are dead. It’s the pharmacies people go to.” (Interview Participant-2, M).

Primary healthcare centres were preferred for affordability but often lacked medicines, hospitals were viewed as a last resort due to cost.

The financial barrier was a recurring theme, with out-of-pocket payments often limiting access. Yet, participants also expressed personal responsibility, with some saying: *“No matter the cost, I’ll borrow to treat my children” (Interview Participant-8, F).*

### Public Perceptions of AMR Responsibility and Suggestions for Change

While 834 (65.11%) believed antibiotics are effective for treating infections, fewer 376 (29.35%) thought AMR is a serious public health issue in their community. In contrast, 630 (49.18%) were unsure whether AMR is a serious issue, and 275 (21.47%) denied its seriousness altogether. Responsibility for AMR was primarily attributed to healthcare providers 811 (63.36%) and government 656 (51.25%). Only 212 (16.56%) of participants believed individuals have a role in combating AMR. This outsourcing of responsibility suggests that many do not recognize their own behaviour as contributing to the AMR problem, a significant obstacle to community-level behaviour change. Participants in the interviews and focus groups proposed multifaceted solutions to antibiotic misuse. They called for improved public health education, community engagement, clearer guidance from healthcare providers, and stronger government intervention. Strategies such as radio jingles, posters, local language film, visual campaigns, and grassroots outreach were recommended:

*“Radio jingles will work... some people in the villages won’t see billboards.” (Interview 18)*

*“Sensitization is key. Even at pharmacies, they should tell you to finish your dose once you start.”* (Interview 1).

The role of religious and community leaders was emphasised, along with the need for individual accountability:

*“Change begins with me. If I do the right thing, others might follow*.” (FGD 2)

There was consensus that interventions must be culturally embedded, locally led, and sustained. Some participants suggested leveraging schools, churches, and marketplaces as platforms for education. The statement above “change begins with me” was echoed in several discussions, revealing a willingness to adopt personal responsibility if adequately supported.

*“If you want to live, you’ll follow the advice. It’s very easy. The problem is people don’t know why it matters.”* (Interview 1). However, this willingness can only be realised if structural constraints, cost, access, and weak regulation, are addressed alongside education.

### Structural and contextual challenges

Antibiotic misuse appeared to be connected to structural barriers shaping health access. Poverty, lack of health insurance, weak regulation frameworks, and overburdened health facilities were significant motivators of inappropriate antibiotic behaviour. These emerged as key enablers of antibiotic misuse. Respondent accounts speak to the kind of limited oversight of antibiotic dispensing reported in the literature; participants reported that prescriptions were rarely required:

“*Antibiotics are over the counter. You don't need a prescription*.” (Interview 1).

*“We don’t have the capacity for everyone to go to hospital for malaria [and other diseases]. Hospitals are for the rich.” (Interview Participant-2, M).*

The inadequacy of public healthcare services was also evident, with one FGD participant stating: *(“Health centres are dead. If you want real care, you go to the private hospitals, if you can afford it.”).*

Pharmacies and chemist shops were trusted but often functioned as both diagnostic and treatment centres, especially in communities where doctors were inaccessible. In some cases, participants reported that chemists sometimes overstepped their roles, while in others, their advice was crucial. Participants distinguished between pharmacies and “patent medicine dealers,” the latter seen as less trained but more widely accessible:

*“Chemists are mostly not skilled. They are not professionals. But that is where people go.” (Interview 1).*

## Discussion

This study provides an examination of antimicrobial drug use patterns, knowledge of AMR, and motivations behind antibiotic misuse among communities in Enugu, Nigeria. By combining large-scale survey data with qualitative insights, the findings offer critical reflections on how community members understand, access, and deploy antibiotics in everyday life. Through the baseline survey complemented by interviews and focus group discussions (FGDs), this study attempts to offer community-level findings of antibiotic use in South-Eastern Nigeria. While prior studies have identified high rates of self-medication and misconceptions about antibiotics in other settings like Eritrea, Ethiopia, and East Africa [22–24], this study extends those findings by presenting evidence of under-reported instances of self-medication with antibiotics, including their use as an attempted means to terminate pregnancy, and for other unsanctioned purposes, such as preventive “cleansing” to ward off illness. These practices, like other forms of unprescribed antibiotic use, contribute to pressure on bacteria, thereby driving the development of AMR. Additionally, this study contributes to AMR scholarship by highlighting how antibiotic misuse is shaped not only by limited awareness, but also by persistent structural barriers to healthcare and entrenched patterns of informal care-seeking. Importantly, while it may seem as though some global discourses on AMR often characterise community behaviours as irrational or maybe uninformed, our study findings suggest that these practices are sometimes rational responses by community members to systemic healthcare failures amongst other reasons. Overall, the evidence reveals a complex interplay between health beliefs, socioeconomic pressures, structural inadequacies, and deep-seated cultural practices that continue to shape the misuse of antibiotics in ways that demand urgent, context-sensitive responses.

### Widespread antibiotic misuse and the persistence of non-evidence-based practices

A notable finding of this study is the high prevalence of inappropriate antibiotic use. More than half of all respondents reported using antibiotics in the past six months, with nearly 50% doing so without consulting a healthcare professional. This figure echoes earlier estimates of non-prescription antibiotic use in Nigeria [7] and more broadly in other LMICs [8]. Our findings move beyond prevalence to emphasise why and how these behaviours still occur. Economic hardship, limited access to regulated healthcare, and overreliance on chemist shops or informal vendors consistently emerged as key drivers of misuse. Also, our findings highlight the extent of confusion about the nature, causes, and appropriate treatment of illness. Therefore, there is need for interventions that address both knowledge gaps and the everyday constraints shaping antibiotic use.

Our finding that antibiotics were commonly used for a wide range of conditions such as malaria, fever, and cough, ailments often caused by viral or parasitic pathogens against which antibiotics are ineffective is consistent with other studies, including Zhang et al. [31]. Broader patterns of antibiotic misuse and limited awareness have similarly been reported in Eritrea [22] and Ghana [15], highlighting the need to recognize that presently, antibiotics use by the lay public in many communities is not guided by biomedical understandings of their nature and effects. And therefore, the public needs a continuous better, basic, biomedical understanding of disease and drugs. It is important to strengthen basic biomedical literacy around disease and drug action. However, our findings add critical insight: even when community members possessed partial knowledge about antibiotics, this was frequently overridden by their practical realities (which are): what they could afford, access, or the fact that they trust antibiotics to ‘always’ work. At the same time, this trust was fragile and sometimes contradicted by personal experience. Taken together, the findings suggest that health decision making is based more on hope, trust and anecdotal knowledge than a more informed understanding of health, disease and drugs. While 70% of our study participants felt antibiotics were effective in their recent experience, it is possible that some of these perceived recoveries may have occurred without any treatment or were influenced by a placebo effect, particularly where antibiotics were used for self-limiting conditions. The participants symptom improvement may not have been due to antibiotic action, especially given the high proportion of use for illnesses like malaria or viral infections where antibiotics are not indicated

### Antibiotic use for non-medical and reproductive purposes

Perhaps our most novel and concerning finding is the repurposing of antibiotics for inappropriate uses, particularly in efforts to ‘terminate’ unwanted pregnancies. The accounts that some women take high doses of antibiotics often in combination with strenuous physical activity to “flush out” pregnancies (Participant: FGD 1), signals a dangerous public health blind spot. The use of the term “flush” warrants closer anthropological and biomedical attention. In participants’ accounts, the term “flush” appeared to convey notions of bodily cleansing and flow/flushing, suggesting that these ideas may shape local understandings of health, reproduction, and the body. It is plausible that severe side effects from antibiotic overdose (e.g., nausea, vomiting, diarrhoea, and abdominal cramping) are interpreted as evidence of the body purging, that is flushing of body contents and fluids, which might tally with their local views on cleansing and health. This, in turn, could reinforce the belief in antibiotics effectiveness. From a biomedical perspective, while antibiotics are not an accepted or recognized as abortion-inducing drugs, some studies have shown that exposure to certain antibiotics during early pregnancy have been associated with increased risk of miscarriage [32–34], this may help explain the perceptions by some participants that the practice of taking antibiotics in high doses to terminate pregnancy ‘works’ for some women. However, this belief and practice carry profound risks, high-dose and inappropriate use of antibiotics could accelerate AMR, and also cause organ toxicity, particularly to the liver and kidneys, given the high metabolic load of overdose (in this case antibiotics) and there is the risk of immune system weakening. This practice of using antibiotics with the intention of terminating an existing pregnancy, is not only medically unfounded (antibiotics are neither abortifacients nor contraceptives) but also shows how antibiotics have become entangled with reproductive anxieties, gendered stigma, and unmet sexual and reproductive health needs, particularly in settings where abortion is legally restricted, and access to safe reproductive care is limited. The emergence of this form of misuse in this study highlights the urgent need for integrated approaches that link AMR education/awareness with sexual and reproductive health rights (SRHR). Public health campaigns that overlook this intersection, could risk being ineffective or, worse, complicit in perpetuating silent harms.

### Faith, familiarity, and the cultural logic of illness

Participants frequently described illness using familiar or faith-based frameworks “sore throat,” “infection,” “normal fever,” or even “spiritual attack.” Healing was occasionally attributed to prayer, with antibiotics acting as supplementary rather than curative interventions. These narratives could complicate biomedical framings of drug resistance by showing that health behaviours are not only influenced by knowledge but also by cultural interpretation and spiritual belief [35,36]. Maybe as a way further, rather than viewing such beliefs as barriers, future AMR interventions could recognise them as alternative logics that coexist with biomedical reasoning, shaping what actions feel appropriate and when. Many of the health-seeking behaviours whether prayer, antibiotics, or even herbal remedies are perhaps sometimes addressing illnesses that would have resolved on their own due to the body’s immune system, and not necessarily because of the chosen treatment. Understanding that our immune system could be doing its work is as important as acknowledging other parallel belief systems or treatment.

### Chemists as gatekeepers and low awareness of AMR

The role chemists and patent medicine vendors play in Nigeria is quite ambivalent, that is often playing two opposing roles, because they are somewhat both vital and risky. On one hand, they serve as the most accessible and trusted source of care to many, especially in under-resourced areas where hospitals are seen as expensive, distant, or ineffective. On the other hand, these informal providers seem to operate ignoring regulatory oversight [7,10,24]. Participants’ accounts suggested that chemists often frequently dispensed antibiotics without prescriptions and sometimes mixed drugs in dangerous combinations either due to customer demand, financial cost, or limited knowledge. These double roles chemists play as both lifeline and liability highlights findings from Tanzania and Uganda during COVID-19 [4] where drug sellers often dispensed antibiotics without prescription. Additionally, this study finds a severe lack of awareness about AMR, only 15% of respondents had heard of the term, and among those, most misunderstood it. Blame was frequently displaced onto “fake drugs” or the healthcare system, with only 16% acknowledging individual responsibility for curbing resistance. This reinforces previous findings that link AMR with abstract, distant threats rather than immediate, personal consequences [37]. Effective interventions must, therefore, make AMR relatable and local grounded in people’s everyday decisions and tied to personal risk and communal harm.

### Structural and health system failure

While this study predominantly identified individual beliefs and behaviours shaping antibiotic use, participants’ responses also hinted at broader structural problems of system failures, including gaps in care, insurance, public education, and safe treatment pathways. For example, participants’ behaviours, including premature discontinuation of treatment and reliance on “mixed” drugs, were often not a matter of choice but were born out of constraining factors such as structural and financial constraints. As one participant explained, “the money that you have” determines whether you can access full treatment or a handful of mixed capsules. Such findings resonate with other critiques regarding AMR, which argue that focusing narrowly on behaviour change obscures the political economy of access and inequality [38]. Interventions must therefore address healthcare affordability, infrastructure, and regulation, not just knowledge gaps.

Amid these challenges, community members themselves identified and called for grounded solutions such as, radio jingles, religious and market-based outreach, visual campaigns in local languages, co-creation with community members and the involvement of respected figures such as pharmacists and pastors. Religious leaders, market women, and even informal drug vendors play key roles in shaping health behaviours and should be part of the solution. In response to questions about responsibility for AMR and potential solutions, some participants echoed the idea that “change begins with me,” revealing an openness to accountability when appropriately informed and supported. These insights validate call for participatory approached to AMR education and interventions that do not impose top-down behaviour change but instead co-create strategies with those most affected [2], an approach that is central to other phases of this research.

Overall, this study provides a grassroots-level analysis of antibiotic use and resistance in a Sub-Saharan African setting. Contributing novel insights into unregulated antibiotic practices, socially embedded misconceptions, and emerging non-medical uses of antibiotics. Given the global nature of AMR, these local dynamics could have far-reaching implications for national policy, international funding priorities, and the design of AMR community engagement strategies in LMICs. The findings reveal the urgency of investing in health literacy, strengthening regulatory mechanisms, and improving healthcare access in ways that address not only structural barriers but also everyday lived realities. Additionally, the study contributes to future participatory action research for AMR community engagement by highlighting locally relevant, culturally appropriate, and socially inclusive patterns that could be used in future to promote adequate antibiotic use.

### Strengths and limitations

One of the strengths of this study lies in its mixed-methods design, which integrated quantitative data from a large and representative survey (n = 1,281) with qualitative insights drawn from interviews and focus group discussions. This enabled first the identification of broad patterns of antimicrobial use behaviours and misuse across communities, before then allowing for a more nuanced understanding of the socio-cultural dynamics underpinning antibiotic decision-making in Nigeria. This study captures community-level behaviours, providing a grassroots perspective that is essential for designing effective public health interventions. Again, the study’s geographical and demographic coverage is notable when compared with many existing studies in Nigeria, which often focus on specific groups such as students or patients in healthcare facilities. It included participants from all the local government areas within the study location, spanning both rural and urban settings, and accounted for variation in age, gender, education, and employment. This broad coverage could strengthen the generalisability of the findings within the context of South-Eastern Nigeria and reflects real-world patterns of antibiotic access and use. However, comparative research in other states and countries is needed to assess broader applicability. Another significant strength of this study is its deliberate inclusion of persons with disabilities (PWDs), a group often marginalised in AMR research and interventions. While the study did not collect sufficient data to analyse their experiences in detail, ensuring that PWDs were represented in the sample reflects a sensitivity to their significance as a subgroup in the population. Their participation meant that they were not excluded from recruitment, and future research should build on this by engaging more deeply with the specific experiences and needs of PWDs in relation to antibiotic use and AMR. The study also uniquely identifies emerging and under-reported practices, such as the repurposing of antibiotics for non-medical purposes such as preventive “cleansing” and attempted pregnancy termination. These critical, novel findings thereby expand academic understanding of lay antibiotic use and of community practices that may contribute to the development of AMR.

Despite its strengths, the study is not without limitations. First, self-reported data were used in both the survey and qualitative methods, which may be subject to recall bias or social desirability bias. Participants may have over- or under-reported their use of antibiotics or awareness of AMR, especially in a context where some drug practices (e.g., unprescribed use or abortion-related use) carry stigma or legal risk. Second, although the study design and cooperation with local partners enabled a large sample spread across all LGAs of Enugu State, the current data analysis is descriptive, not yet exploring complex statistical associations. Third, while the study recognised the importance of not excluding persons with disabilities, the data collection and analysis did not enable significant insights into the specific experiences of this subgroup. Nor was it possible to disaggregate findings within this population (e.g., adults vs. children, men vs. women, physical vs. mental disabilities, visible vs. hidden conditions, or levels of dependence). Future research should seek to address these important within-group distinctions to better capture the diversity of experiences among people living with disabilities. Future research should prioritise understanding the scale, drivers, and health consequences of this form of misuse, particularly its intersection with gender, stigma, and unmet reproductive health needs. Evidence from such work could inform integrated public health interventions that address AMR while safeguarding sexual and reproductive health. Further research is needed to examine the availability, content, and community-level impact of existing AMR-related public health materials. Also, studies should investigate participatory approaches to public health communication by exploring how communities themselves would frame, co-produce and communicate AMR messages.

## Conclusion

This study provides an in-depth, grassroots-level understanding of antimicrobial drug use and misuse in Enugu State, Nigeria. It highlights the complex interplay of knowledge, culture, access, and structural inequities that underpin antibiotic practices in everyday life. The findings reveal that antibiotic misuse is not merely a product of ignorance or indifference, but is deeply embedded in economic constraints, sociocultural norms, and healthcare limitations. Across both the survey and qualitative findings, participants reported frequent antibiotic use for conditions such as malaria, cough, and typhoid regardless of the biomedical appropriateness of such treatments. Practices such as obtaining antibiotics from unregulated chemists, discontinuing treatment prematurely, and using antibiotics without prescriptions were common, which corresponds with several existing studies in Nigeria and Sub-Saharan Africa. This study advances the filed by documenting novel and unreported alarming practices which is the use of antibiotics for non-medical purposes, including self-managed pregnancy termination and so-called preventive “cleansing” highlighting the intersection of antimicrobial misuse with broader reproductive, gendered, and health system gaps. The study also highlights the limited public understanding of antimicrobial resistance, with only a small proportion of participants aware of the concept, and even fewer possessing accurate knowledge. While blame was often directed toward healthcare providers or pharmaceutical companies, personal responsibility was rarely acknowledged, suggesting a critical disconnect between public health messaging and local perceptions of accountability. While participants did not explicitly call for co-produced interventions, their suggested solutions such as locally led education campaigns, faith-based outreach, and stricter regulation, reflect priorities that could be effectively realised through inclusive and collaborative, community co-production approaches.

## Supporting information

S1 Text Final questionnaire. (PDF)

S1 Checklist Inclusivity in Global Research. (DOCX)

S1 Appendix Final Nvivo codebook. (PDF)

S1 Table Full detailed survey data tables. (PDF)
