# Supplementary material for: Understanding community antibiotic use and antimicrobial resistance in Sub-Saharan Africa: A grassroots perspective from Enugu, Nigeria
Source: PLoS One. 2026 Jul 23;21(7):e0353762. doi: 10.1371/journal.pone.0353762 (PMC13395351; doi:10.1371/journal.pone.0353762)
Supplement: S1 Text — (PDF) [file pone.0353762.s001.pdf]

Baseline Survey Questionnaire on Antimicrobial Drug Use, Knowledge, Attitudes, and Behaviours in Enugu Nigeria

Demographic Information

1. Age: ______
2. Gender: Male / Female / Other ______
3. Education Level: [ ] None [ ] Primary [ ] Secondary [ ] Tertiary [ ] Other (specify) ______
4. Occupation: ______
5. Marital Status: [ ] Single [ ] Married [ ] Divorced [ ] Widowed [ ] Other (specify) ______
6. LGA (Please specify the community/town/village you live in Enugu): ______

Section 1: Antibiotics Drug

1. Do you know what antibiotics are? [ ] Yes [ ] No
2. Do you think antibiotics can treat (select all that apply):

   (a) cough

   (b) diarrhoea

   (c) malaria

   (d) headache

   (e) gonorrhoea

   (f) Tuberculosis

3. Which of these do you think best describes antibiotics (select all that apply):

   A. Antibiotics are medications that kill or inhibit the growth of bacteria.

   B. Antibiotics are medications used to treat viral infections.

   C. Antibiotics are medications that can treat any sickness (including stubborn ones).

   D. Antibiotics are medications that reduce inflammation and pain.

(Definition: Antibiotics are medications used to treat bacterial infections by either killing bacteria or preventing their growth and reproduction).

4. Based on the picture drug card shown above, have you used any of them in the last 6 months? [ ] Yes [ ] No

Section 2: Antibiotics Drug Use Practices

5. Have you taken antibiotics in the past six months? [ ] Yes [ ] No
    - If yes, for what purpose? (e.g., illness, preventive, self-medication)
    - What do you normally use drugs like this for? (what sickness).

6. Where do you usually obtain antibiotics?
    - Prescribed by a doctor
    - Purchased from a pharmacy without a prescription
    - Chemist shops
    - Obtained from family or friends
    - Other (specify) ______

7. In what quantity do you obtain antibiotics?
    - Enough for 5-7 days or just a few days worth
    - Other (specify) ______

8. At what point do you stop taking antibiotics for an illness?
    - Once you feel fine
    - Once you have completed the dosage, the doctor or pharmacist advise
    - Once the dosage you could afford finishes, even if you don't feel well enough
    - Other (specify) ______

9. In the past six months, have you taken antibiotics and felt like they did not cure you?

   [ ] Yes

   [ ] No

- If yes, please describe what you did after you noticed it did not cure you (e.g. did you take stronger antibiotics)?

Section 3: Attitudes towards Antibiotic Use and Antimicrobial Resistance (AMR)

1. Have you heard of antimicrobial resistance (AMR)/drug resistance? [ ] Yes [ ] No

   If yes, please explain your understanding:

Definition: Antimicrobial resistance (AMR) refers to the ability of microorganisms (such as bacteria, viruses, fungi, and parasites) to resist the effects of medications previously used to treat them, rendering these medications ineffective in combating infections.

2. How concerned are you about the growing problem of antimicrobial resistance?

   1 – Not concerned

   2 – Slightly concerned

   3 – Moderately concerned

   4 – Very concerned

   5 – Extremely concerned

3. Do you think antibiotics are effective in treating infections? [ ] Yes [ ] No [ ] Not Sure
4. Do you think antimicrobial resistance is a serious public health issue in your community?

   [ ] Yes

   [ ] No

   [ ] Not Sure

5. Who do you think is responsible for addressing the issue of antimicrobial resistance? (select all that apply)
   - Government
   - Healthcare providers
   - Pharmaceutical companies
   - Individuals

- Other (specify) ______

Section 4: Motivations for Antibiotic Misuse

1. Have you ever taken antibiotics without consulting a healthcare professional? [ ] Yes [ ] No

   If yes, what motivated this decision (factors that influence your decision to take antibiotics without prescription)?

Section 5: Additional Comments

Is there anything else you would like to share about your experiences with antibiotics or antimicrobial resistance?
