# Supplementary material for: Understanding community antibiotic use and antimicrobial resistance in Sub-Saharan Africa: A grassroots perspective from Enugu, Nigeria
Source: PLoS One. 2026 Jul 23;21(7):e0353762. doi: 10.1371/journal.pone.0353762 (PMC13395351; doi:10.1371/journal.pone.0353762)
Supplement: S1 Appendix — (PDF) [file pone.0353762.s003.pdf]

FGD and Interviews (AMR Project)

Codes\\Final Codes for FGD and Interviews

Name	Description	Files	References
Antibiotic Use and Misuse	Participants behaviours related to the use and misuse of antibiotics. Including how, motivations, why, and for what illnesses antibiotics are obtained and used, often without prescriptions or professional guidance.	3	20
Easy access without prescription		2	2
High trust in antibiotics; seen as “strong medicine”		2	5
Mixing antibiotics with other drugs by chemists		1	9
Use for non-medical reasons (e.g. attempted pregnancy termination)		2	2
Use of antibiotics for viral or unknown causes		1	2
Knowledge and Misconceptions	Captures participipnats understanding, beliefs, and misunderstandings about antibiotics and AMR. References to how they define antibiotics, what they believe antibiotics can treat, and their interpretations of resistance.	1	8

Name	Description	Files	References
Confusion about appropriate usage and dosage		1	2
Lack of awareness of antibiotic resistance and its causes		1	1
Limited understanding of what antibiotics do		1	3
Misidentification of antibiotics (e.g. cod liver oil)		1	2
Perceptions and Experiences of Illness	Includes participants' understandings, interpretations, and lived experiences of illness in Enugu Nigeria. Participants description of illenses and common symptoms (e.g., cough, sore throat, fever, shortness of breath). Etc	1	14
Confusion between respiratory infections, malaria, and typhoid		1	3
Faith-based interpretations (e.g. healing through prayer)		1	7
Familiar symptoms attributed to malaria or general sickness		1	2
Local names and explanations for illnesses		1	2

Name	Description	Files	References
Structural and Contextual Challenges	Systemic, social, and economic conditions that shape antibiotic use and misuse within communities. It	2	8
Inadequate primary healthcare infrastructure		1	1
Lack of regulation and enforcement on antibiotic sales		1	2
Lack of trust or dissatisfaction with hospitals		1	2
Poverty and inability to afford tests or full treatment		1	3
Suggestions for Change and Community Education	Recommendations for addressing antibiotic misuse and improving awareness of AMR at the community level.	2	13
"Change begins with me" – individual-level commitment		1	1
Flyers, posters, t-shirts, short films, and community campaigns		1	3
Role of government, subsidies, regulation enforcement		1	7

Name	Description	Files	References
Role of religious, community institutions in awareness		1	1
Treatment-Seeking Behaviours	How, when, and where participants seek treatment when they become ill.	3	36
Delay due to cost, time, or assumptions of self-recovery		2	9
First-line treatment self-medication, local herbs (agbo), chemists		3	19
Health centers seen as more affordable than hospitals, but often lacking drugs		1	5
Recourse to private hospitals only after other options fail		1	3
