# Supplementary material for: Understanding community antibiotic use and antimicrobial resistance in Sub-Saharan Africa: A grassroots perspective from Enugu, Nigeria
Source: PLoS One. 2026 Jul 23;21(7):e0353762. doi: 10.1371/journal.pone.0353762 (PMC13395351; doi:10.1371/journal.pone.0353762)
Supplement: S1 Table — (PDF) [file pone.0353762.s004.pdf]

Detailed Baseline Survey Data Table -Supplementary Material

Response	Count	Percentage (%)
**Age Group**		
18-25	54	4.22
26-35	127	9.91
36-45	327	25.53
46-55	353	27.56
56 and above	420	32.79
**Persons with Disability**		
Yes	128	9.99
No	1153	90.01
**Education Level**		
Postgraduate education	20	1.56
Undergraduate education	184	14.36
No formal education	253	19.75
Primary school	356	27.79
Secondary school	468	36.53
**Employment Status**		
Self-employed	764	59.64
Unemployed	293	22.87
Retired	86	6.71
Employed (full-time)	78	6.09
Employed (part-time)	48	3.75
Student	12	0.94

**Antibiotics Misconception Analysis**		**Count**	**Percentage (%)**
Do you think antibiotics can treat: (Cough)	False	580	60.67
Do you think antibiotics can treat: (Cough)	True	376	39.33
Do you think antibiotics can treat: (Diarrhea)	False	748	78.24
Do you think antibiotics can treat: (Diarrhea)	True	208	21.76
Do you think antibiotics can treat: (Malaria)	True	617	64.54
Do you think antibiotics can treat: (Malaria)	False	339	35.46
Do you think antibiotics can treat: (Headache)	False	778	81.38
Do you think antibiotics can treat: (Headache)	True	178	18.62
Do you think antibiotics can treat: (Gonorrhoea)	False	598	62.55
Do you think antibiotics can treat: (Gonorrhoea)	True	358	37.45
Do you think antibiotics can treat: (Tuberculosis)	False	808	84.52
Do you think antibiotics can treat: (Tuberculosis)	True	148	15.48

**Description**	**Count**	**Percentage (%)**
Antibiotics are medications that kill or inhibit the growth of bacteria	452	47.03
Antibiotics are medications used to treat viral infections	327	34.03
Antibiotics are medications that can treat any sickness (including stubborn ones)	151	15.71

Antibiotics are medications that reduce inflammation and pain	31	3.23
**Purpose of Antibiotics Use in the last six month**		
**Purpose Category**	**Count**	**Percentage (%)**
Illness/Treatment	470	75.32
Self-medication	68	10.9
Preventive	68	10.9
Other	18	2.88

**What sickness do you use antibiotics to treat?**	
**Illness**	**Count**
malaria	239
typhoid	128
infection	89
cough	56
fever	33
sickness	23
illness	18
pain	15
headache	14
wound	10
catarrh	6
rashes	6
pregnancy	5
injury	5
bacteria	5
diarrhea	4
skin	4
boil	3
gonorrhea	3
weakness	3
itching	3
injuries	3
growth	2
ulcer	2
vomiting	2
std	2
pneumonia	2
eye	1
ear	1
meningitis	1
stroke	1
arthritis	1

**Source of antibiotics**	**Count**	**Percentage (%)**
Chemist shops	804	62.76
Purchased from a pharmacy without a prescription	212	16.55

Prescribed by a doctor	193	15.07
Other (please specify)	57	4.45
Obtained from family or friends	15	1.17
**At what point do you stop taking antibiotics for an illness?**		
**Response**	**Count**	**Percentage (%)**
Once you feel fine	311	49.84
Once you have completed the dosage, the doctor or pharmacist advises	253	40.54
Once the dosage you can afford finishes, even if you don't feel well enough	56	8.97
Other (please specify)	4	0.64
**what you did after you noticed [that Antibiotics you took] it did not cure you**		
**Response Category**	**Count**	**Percentage (%)**
Other	1135	88.6
Took stronger/different antibiotics	66	5.15
Visited health facility	29	2.26
Used herbal or traditional remedy	22	1.72
Did nothing	14	1.09
Returned to chemist/pharmacist	13	1.01
Experienced adverse reaction	2	0.16
**(factors that influence your decision to take antibiotics without prescription)?**		
**Theme**	**Count**	**Percentage (%)**
Illness / Symptoms	216	34.78
Financial Constraints	152	24.48
Accessibility Issues	133	21.42
Influence of Others	59	9.5
Self-Diagnosis / Confidence	51	8.21
Time and Convenience	34	5.48
Ignorance / Misinformation	21	3.38
